# Temporal relationship of computed and structured diagnoses in electronic health record data

**DOI:** 10.1186/s12911-021-01416-x

**Published:** 2021-02-17

**Authors:** Wade L. Schulz, H. Patrick Young, Andreas Coppi, Bobak J. Mortazavi, Zhenqiu Lin, Raymond A. Jean, Harlan M. Krumholz

**Affiliations:** 1 Department of Laboratory Medicine, Yale School of Medicine, New Haven, CT USA; 2 Center for Outcomes Research and Evaluation, Yale New Haven Hospital, New Haven, CT USA; 3 Section of Cardiovascular Medicine, Department of Internal Medicine, Yale School of Medicine, New Haven, CT USA; 4 Department of Computer Science and Engineering, Texas A&M University, College Station, TX USA; 5 Center for Remote Health Technologies and Systems, Texas A&M University, College Station, TX USA; 6 Department of Surgery, Yale School of Medicine, New Haven, CT USA; 7 Department of Health Policy and Management, Yale School of Public Health, New Haven, CT USA

**Keywords:** Real-world data, Electronic health records, Observational studies, Computational phenotypes

## Abstract

**Background:**

The electronic health record (EHR) holds the prospect of providing more complete and timely access to clinical information for biomedical research, quality assessments, and quality improvement compared to other data sources, such as administrative claims. In this study, we sought to assess the completeness and timeliness of structured diagnoses in the EHR compared to computed diagnoses for hypertension (HTN), hyperlipidemia (HLD), and diabetes mellitus (DM).

**Methods:**

We determined the amount of time for a structured diagnosis to be recorded in the EHR from when an equivalent diagnosis could be computed from other structured data elements, such as vital signs and laboratory results. We used EHR data for encounters from January 1, 2012 through February 10, 2019 from an academic health system. Diagnoses for HTN, HLD, and DM were computed for patients with at least two observations above threshold separated by at least 30 days, where the thresholds were outpatient blood pressure of ≥ 140/90 mmHg, any low-density lipoprotein ≥ 130 mg/dl, or any hemoglobin A1c ≥ 6.5%, respectively. The primary measure was the length of time between the computed diagnosis and the time at which a structured diagnosis could be identified within the EHR history or problem list.

**Results:**

We found that 39.8% of those with HTN, 21.6% with HLD, and 5.2% with DM did not receive a corresponding structured diagnosis recorded in the EHR. For those who received a structured diagnosis, a mean of 389, 198, and 166 days elapsed before the patient had the corresponding diagnosis of HTN, HLD, or DM, respectively, recorded in the EHR.

**Conclusions:**

We found a marked temporal delay between when a diagnosis can be computed or inferred and when an equivalent structured diagnosis is recorded within the EHR. These findings demonstrate the continued need for additional study of the EHR to avoid bias when using observational data and reinforce the need for computational approaches to identify clinical phenotypes.

## Background

Despite the rapid digitization of healthcare, the current research enterprise remains inefficient. Randomized control trials (RCTs), which remain the gold standard, are costly, time-consuming, and capture only a small cross-section of patients, which limits their generalizability [1, 2]. To accelerate the pace of discovery, investigators and regulatory agencies have increasingly focused on real-world data (RWD), defined as data collected outside of a traditional research environment, as a source of information [3–5]. Real-world data sources include administrative claims and discharge databases, clinical registries, and electronic health records (EHRs), among others. Despite increased access to these digital data, it remains important for investigators to be aware of and account for limitations in these observational data sets during study design and analysis [6–9].

The creation of patient cohorts and study endpoints often requires the identification of clinical diagnoses. These populations and endpoints are frequently characterized by diagnostic codes, such as International Statistical Classification of Diseases and Related Health Problems 10^th^ Revision Clinical Modification (ICD-10-CM) codes [10]. Observational and outcomes research have long used administrative claims and registries as a source of this information [11–18]. However, these repositories come with the known limitations of significant time delays in availability and a lack of detailed clinical records [19, 20]. In addition, significant costs are associated with manual abstraction for disease-specific registries [21]. Because of these limitations and the increased access to detailed EHR data, investigators have increasingly focused on the EHR to provide the data needed to support a wide range of studies.

Information obtained from the EHR has the potential to provide near real-time access to a more complete data set than can be provided from other RWD sources [19, 20]. The EHR is the primary repository of a patient's clinical history, but the data elements needed to represent a patient's history can be found in many locations, from structured fields in the history and problem list to unstructured clinical notes [9]. Prior work has shown that patient history and problem lists within the EHR can be incomplete and contain frequent errors [22–25]. Even for a relatively straightforward diagnosis such as hypertension, researchers from the OneFlorida clinical data research network found that as many as 30% of those they identified with hypertension by means of clinical measurements recorded in the EHR were missing the associated structured diagnostic code [26], similar to results found by an earlier study from Stanford [27].

Yet studies based on EHR data frequently use, sometimes solely, structured diagnostic codes to create cohorts and identify outcomes [28, 29]. Since administrative claims are ultimately derived from these structured fields, the EHR is likely not significantly worse than claims-based sources. Investigators have also demonstrated that structured diagnostic codes can provide valuable information when analyzed in the appropriate context [30, 31]. Therefore, while limitations to the use of structured diagnoses from the EHR exist, they remain frequently used and ongoing study can increase the value of results generated from these sources.

A primary goal of EHR-based studies is access to near real-time information [32, 33], but it remains an indirect assessment of a patient's status due to how the EHR is used in clinical workflows [34]. While data may be extracted immediately, clinical workflows and decision making may impact the timeliness of data entry into the EHR, particularly within structured data elements. Hence, the extent to which structured diagnoses may be missing at the time of analysis may be greatly underestimated. Therefore, computed diagnoses may be needed in order provide more accurate and timely information. While no single approach to creating a computed phenotype is necessarily best, understanding the limitations of EHR and other RWD sources is crucial for appropriate study design and interpretation of results.

In this study, we determine how structured diagnostic codes in the patient history and problem list compare to computed diagnoses for hypertension (HTN), hyperlipidemia (HLD), and diabetes mellitus (DM). We selected these three diagnoses with because they can be efficiently computed from structured clinical and laboratory data. We extend on prior work in the field to identify not only the completeness of structured diagnostic codes, but also the temporal association between the computed diagnosis and manual recording of an equivalent diagnostic code within the clinical record.

## Methods

### Data sources

We created our data set from a complete extract of the Yale New Haven Health clinical data warehouse (Epic Caboodle) that was transformed into the PCORnet Common Data Model v3.1 (CDM) on February 11, 2019 using our local data analytics platform [35]. The Caboodle source tables and supporting terminology tables were transformed into the *demographic*, *encounter*, *diagnosis, condition*, *lab_result_cm*, and *vital* PCORnet CDM tables, which were used for analysis. Diagnosis and condition source were categorized as defined in the PCORnet CDM [36] for *OD*, *BI*, and *CL* which we label as *provider*, *billing*, and *claims*. As a data quality study based on existing and deidentified data, this work was not classified as human subjects research and did not require Institutional Review Board approval.

### Phenotype definitions

We collected information to assess HTN, HLD, and DM. We excluded data with dates earlier than January 1, 2012 as these represented a sparse fraction of the raw data with unreliable dates of onset due to their transfer between prior EHR systems. The most recent date of measurement or sample collection included in the analysis was restricted to on or before August 11, 2018, whereas diagnosis events were current up to February 10, 2019, allowing a period of at least 26 weeks (approximately six months) between the most recent measurement or result and the final date available. If multiple measurements were performed on the same day, then the minimum value of the measurement was selected to provide the most restrictive threshold and, consequently, a conservative assessment of the possible disease condition. Only outpatient blood pressure readings, as annotated within the PCORnet CDM, were used, but laboratory results were extracted from all encounter settings. Relevant laboratory results for LDL and A1c were identified by LOINC codes (LDL: ‘13457-7’, ‘18262-6’; A1c: ‘4548-4’) code or internal EHR codes (various and specific to our institution).

We flagged measurements as a 'signal of disease' whenever they exceed a specific threshold. For HTN, a measurement was flagged as a 'signal of disease' if either the minimum systolic reading was ≥ 140 mmHg or the minimum diastolic reading was ≥ 90 mmHg. For HLD, the threshold was an LDL ≥ 130 mg/dL and for DM the threshold was an A1c ≥ 6.5%. For all three conditions, if any two measurements taken at least 30 days apart were found above the threshold (abnormal is high in all three cases), the patient was considered to have a computed diagnosis of disease on the date of the second signal.

For each condition and diagnosis code system, the first 3 or 5 characters of the code string were matched against ICD-10-CM and ICD-9-CM parent codes (Table 1). Patients with a diagnosis present prior to the first signal were flagged as having an existing diagnosis. For patients without a prior diagnosis or computed diagnosis as defined above, the first date a matching diagnosis code (Table 1) was recorded in the EHR, if present, was logged along with its origin (provider-entered billing diagnosis; provider-entered encounter diagnosis or problem list entry; or diagnosis code from returned claims) and the date of the patient’s most recent encounter with the health system. A final data set consisting of the computed signal dates, computed and structured diagnosis dates, date of most recent encounter, and diagnosis origin was used for analysis.

**Table 1:** Diagnosis criteria and ICD-9-CM and ICD-10-CM codes used to determine computed and recorded diagnoses

Condition	Signal and diagnosis criteria
Hypertension	Denominator: Outpatient systolic ≥ 140 or diastolic ≥ 90 (mmHg)
	2 readings ≥ 30 days apart and no prior diagnosis
	Numerator: New diagnosis of any of the following
	ICD-9 groups: 401, 402, 403, 404, 405, 642
	ICD-10 groups: I10, I11, I12, I13, I15, I16, I27, O13
Diabetes	Denominator: A1c ≥ 6.5 (%)
	2 readings ≥ 30 days apart and no prior diagnosis
	Numerator: New diagnosis of any of the following
	ICD-9 groups: 249, 250
	ICD-10 groups: E08, E09, E10, E11, E13
Hyperlipidemia	Denominator: LDL ≥ 130 (mg/dL)
	2 readings ≥30 days apart and no prior diagnosis
	Numerator: New diagnosis of any of the following
	ICD-9: 272.0, 272.2, 272.3, 272.4, 272.9
	ICD-10: E78.0, E78.2, E78.3, E78.4, E78.5, E78.7, E78.8, E78.9

*ICD-9-CM* International Classification of Diseases, Ninth Revision, Clinical Modification, *ICD-10-CM* International Classification of Diseases, Tenth Revision, Clinical Modification, *LDL* Low-density lipoprotein cholesterol

### Data analysis and statistical approaches

Data extraction was done with custom PySpark scripts using Spark (v2.1.0). Preprocessing and summary statistics were performed using the pandas (v0.24.1) and NumPy (v1.16.2) Python libraries. Visualizations were produced with the Matplotlib (v3.0.3) and seaborn (v0.9.0) Python libraries. To model the time to diagnosis, we employed the Kaplan–Meier estimation method for survival analysis using the lifelines (v0.20.0) Python library. All study-specific scripts were reviewed by an independent analyst.

Patients with an existing (prior to first signal) or early (recorded between the first and second signal) diagnosis were excluded from the time to diagnosis analysis. The duration for the survival analysis (equivalent to “survival time”) was the number of days between the date of the second signal and the date of diagnosis. For those who were never diagnosed, it was defined as the number of days between the date of the second signal and the date of the most recent encounter, at which point they were censored due to lack of additional follow-up.

## Results

### Frequency of clinical and computed diagnoses

**Fig. 1:**
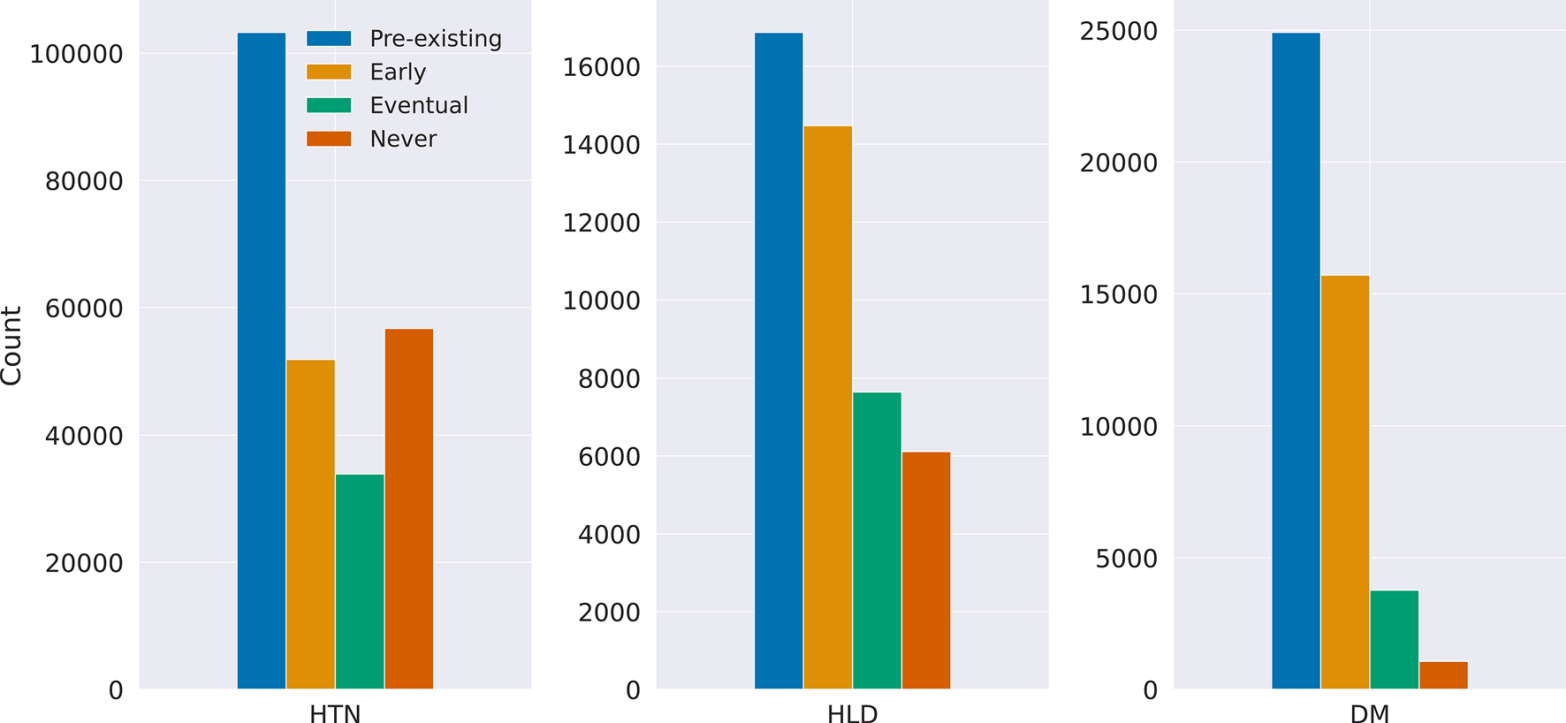
Frequency and temporality of recording a structured diagnosis. For patients with a structured EHR diagnosis, the number of patients who had a pre-existing (preceded the first signal), early (recorded between the first and second signal), or eventual (recorded after the computed diagnosis, or second signal) structured diagnosis recorded and the number of patients who never had a structured diagnosis recorded in the EHR

We defined a signal of disease as an observation above the threshold. In our cohort, we computed a diagnosis of HTN in 245,711 patients, HLD in 45,098 patients, and DM in 45,460 patients who met our criteria for two signals separated by at least thirty days. Of these patients, a pre-existing, structured diagnosis of HTN, HLD, or DM, was present in the EHR for 42.0%, 37.4%, and 54.8% of patients, respectively, before the first signal was identified (Fig. 1). For patients with a new diagnosis, there was a large degree of variability in the presence of structured diagnostic codes among the conditions we assessed. For DM, 76.5% of patients received an early structured diagnosis, meaning a structured diagnosis was recorded in the EHR in the window between the first and second signal. However, for those with HTN or HLD, only 36.4% and 51.3%, respectively, received an early clinical diagnosis. Similarly, 39.8% of those with computed HTN and 21.6% of those with a computed HLD diagnosis never received a structured diagnosis in the EHR, while only 5.2% of those with DM lacked a structured, clinical diagnosis.

### Origin of first clinical diagnosis

**Fig. 2:**
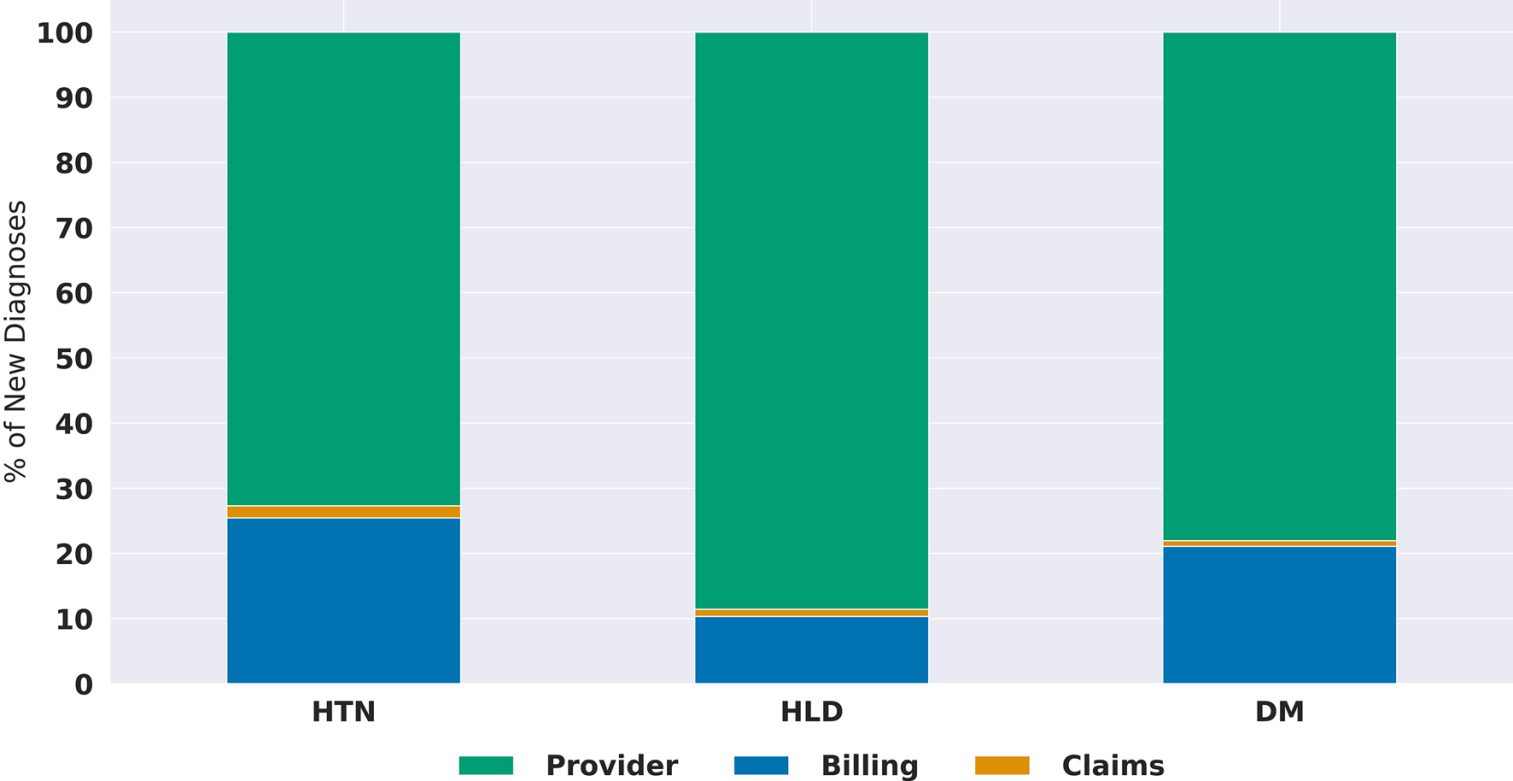
Source of initial diagnosis. The frequency of diagnosis source (provider-entered history or problem list, provider-entered billing, or returned claims) of the first structured diagnosis for patients with a new diagnosis of hypertension (HTN, n = 85,712), diabetes mellitus (DM, n = 19,472), or hyperlipidemia (HLD, n = 22,116)

We also assessed the source of the first structured diagnosis to determine whether it came from a provider-entered, billing, or claims-based diagnosis. For structured diagnoses of different sources that were recorded on the same date, the provider-entered entry took priority, with billing second and claims third in order of precedence. We found that the first source was similar among all three conditions, with most diagnoses being provider-entered within the medical history or problem list, followed by billing-related diagnoses (Fig. 2). Only a small proportion of diagnoses were first identified via returned claims within our local data set.

### Temporality of clinical and computed diagnoses

**Fig. 3:**
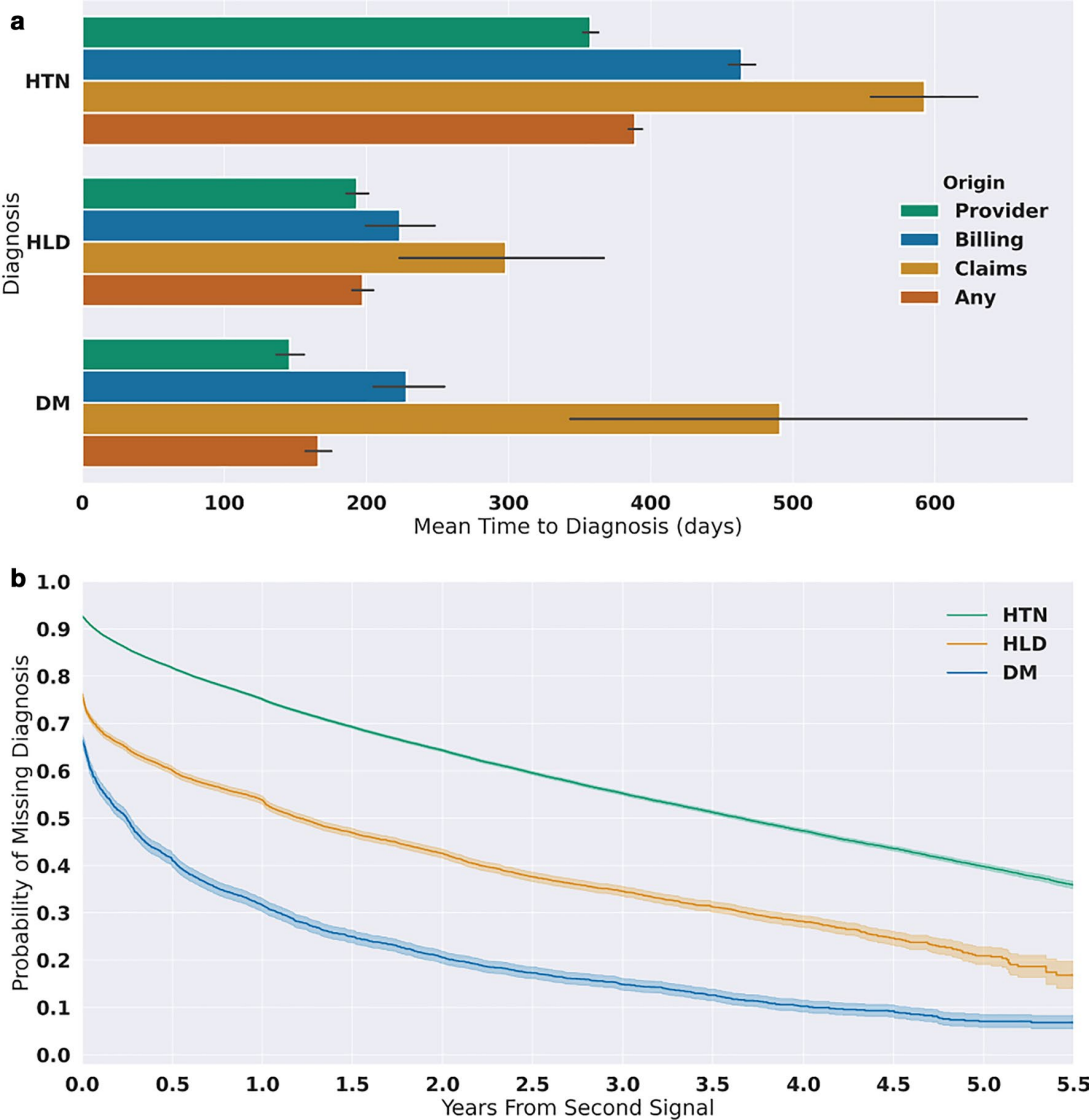
Temporal delay between computed and recorded diagnoses. **a** The mean time in days to receive a structured diagnosis from the time of the second signal. Means were calculated using the number of days to initial diagnosis. **b** Kaplan–Meier estimated probability that a patient is missing a structured diagnosis after a computed diagnosis is made (presence of two signals) for patients with hypertension (HTN, n = 90,614), diabetes mellitus (DM, n = 4836), or hyperlipidemia (HLD, n = 13,754)

Since the timing of diagnosis is relevant to cohort creation, we determined the delay in availability of the first structured diagnosis compared with when a diagnosis could be computed from other data available in the EHR. To define a consistent starting time, patients with a pre-existing diagnosis (present prior to first signal) or early diagnosis (occurring between the first and second signal) were excluded. Within this cohort, the mean time for a structured diagnosis to be recorded ranged from a minimum of 166 days for DM to a maximum of nearly 600 days for HTN (Fig. 3a) from the time of the computed diagnosis. The temporal delay varied by the source of diagnosis, with provider-entered diagnoses having the shortest interval in all conditions and claims-based diagnoses having the longest. It should be noted that very few patients had a claim as the first occurrence of a structured diagnosis (n = 633, 128, and 33 for HTN, HLD, and DM, respectively).

To assess the timeliness of structured diagnoses, we estimated the likelihood of having a structured diagnosis present in the EHR using the Kaplan–Meier estimation method, where the last encounter was used as the censor date if a discrete diagnosis was not found. The likelihood of not having a structured diagnosis also varied by condition (Fig. 3b). While those with DM had nearly an 80% chance of having a structured diagnosis recorded at 2 years, those with HLD had less than a 60% chance and those with HTN had less than a 40% chance of having a structured diagnosis present at the same timepoint. The mean time from the second signal of disease to censoring for those who did not receive a diagnosis was 726 days, 600 days, and 575 days for HTN, HLD, and DM, respectively.

## Discussion

The primary finding of this study was that structured diagnoses within the EHR for HTN, HLD, and DM can have a marked delay in being recorded compared with the time a diagnosis can be computed from other EHR data. For three common diseases, the average time from which a diagnosis could be computed from laboratory values preceded the manual recording of a structured diagnosis by as much as 389 days. In addition, even one year after a diagnosis can be computed, a large percentage of patients do not have an equivalent structured diagnosis recorded in the EHR. Therefore, while the EHR has several potential advantages to other sources of RWD and can be accessed in near real-time from a technical perspective, the recording of clinical information within the structured history and problem list may be less sensitive and delayed compared to identifying computed diagnoses for certain conditions. Thus, studies based on RWD, the approach to extracting information from the EHR may affect its quality.

The EHR contains a detailed record of a patient's clinical history but extracting this history from the structured and unstructured fields that data can reside in remains a challenge. Our work extends the prior literature, which have focused on the completeness and accuracy of the problem list compared to manual adjudication or next generation phenotyping approaches [22, 23, 25, 30, 37, 38]. The delay in recording a structured diagnosis has the potential to impact the development of cohorts and outcome ascertainment from RWD because analyses based on structured diagnostic codes could preferentially identify patients with a longer history of disease. In addition, analyses limited to patients with more recent data are likely to have a lower prevalence of disease than cohorts with a longer history, which may bias historic comparisons with synthetic or external control arms. Finally, if data are obtained from multiple institutions with varying local diagnostic patterns, additional biases may be introduced to multi-site studies.

While concerns of EHR data completeness are often described as data collection and quality issues, this is, in many cases, primarily a concern when the data are used for secondary research purposes [6, 39, 40]. When assessed from a clinical perspective, information related to a disorder, such as blood pressure measurements or documentation within an unstructured clinical note, can be used by a healthcare provider to draw equivalent conclusions, despite the high potential to be missed during automated digital extraction. Therefore, EHR data may not be missing or of low quality, but are rather collected for clinical, rather than research, purposes. Even with these limitations, EHR data can add significant value when analyzed appropriately. For example, others have demonstrated that what may often be described as noise within EHR data, such as frequency of measurements or presence of repeat diagnoses, can actually be used to predict patient outcome and the temporality of clinical conditions [31, 34, 41].

Despite the potential concerns related to the use of EHR described here, it is important to acknowledge that similar issues can also be found in clinical research and manually adjudicated data sets, such as disease registries. Several studies have shown significant variability in the accuracy and inter-rater reliability of manual data abstraction. One case study by the Office of the Inspector General for the Department of Health and Human Services found that manual nurse review identified 78% (93 of 120) of adverse events in the study population [42]. Similarly, patient report, a common source for clinical research studies, has been found to over- or under-represent even major healthcare events, such as readmission, in nearly 30% of cases [43]. Therefore, strategies to better understand and use RWD to augment data collected through traditional methods have the potential to increase the accuracy and completeness of patient history, clinical events, and healthcare outcomes.

This study has several limitations. First, data were collected from a single site within a healthcare system. We did not assess the possible impact of data quality issues in the EHR and its mapping to the PCORnet Common Data Model, a complex issue we consider to be beyond the scope of this study. However, our findings for the number of missing diagnoses for those with HTN were consistent with previously published studies [26, 27]. This work also focused on only three phenotypes which could be reliably identified from clinical measurements and laboratory testing, all of which were chronic diseases, and did not assess for more complex diagnoses or variations in thresholds for diagnosis. Finally, we did not assess the cause or clinical impact of delayed or missing structured diagnoses.

While strategies to assess data quality and account for variations in data collection for clinical research data have been developed, access to and use of RWD remains a new and rapidly evolving field. Like diagnostic laboratory tests, methods to extract data from the EHR can be viewed as assays with varying sensitivity, specificity, and window periods. Work by the Electronic Medical Records and Genomics (eMERGE) [44] and OHDSI [38, 45] networks, among others, to create standardized next generation phenotypes will continue to improve our ability to identify clinical cohorts and outcomes. While no single approach may be effective for every study, standardized strategies to assess whether RWD and specific computed phenotypes are fit-for-purpose will need ongoing, and likely use case-specific, assessment.

## Conclusions

We found that the recording of structured diagnoses within the EHR had a marked time delay compared to when a computed diagnosis could be extracted from clinical findings and laboratory results within the EHR. The delay and presence of a structured diagnosis varied by disease. These findings highlight the need for continued assessment of RWD analysis and the validation of EHR data when used for biomedical research.

## Data Availability

The data sets analysed during the current study are considered sensitive and proprietary and hence are not publicly available. Further details are available from the corresponding author on reasonable request.

## Abbreviations

EHR: Electronic health record
HTN: Hypertension
HLD: Hyperlipidemia
DM: Diabetes mellitus
RCT: Randomized control trial
ICD-10-CM: International Statistical Classification of Diseases and Related Health Problems, 10th Revision Clinical Modification
CDM: Common data model
